# The Chorioallantoic Membrane Xenograft Assay as a Reliable Model for Investigating the Biology of Breast Cancer

**DOI:** 10.3390/cancers15061704

**Published:** 2023-03-10

**Authors:** Raphela A. Ranjan, Julienne K. Muenzner, Philipp Kunze, Carol I. Geppert, Matthias Ruebner, Hanna Huebner, Peter A. Fasching, Matthias W. Beckmann, Tobias Bäuerle, Arndt Hartmann, Wolfgang Walther, Markus Eckstein, Ramona Erber, Regine Schneider-Stock

**Affiliations:** 1 Experimental Tumor Pathology, Institute of Pathology, University Hospital Erlangen, Friedrich-Alexander-Universität Erlangen-Nürnberg, 91054 Erlangen, Germany; 2 Institute of Pathology, University Hospital Erlangen, Friedrich-Alexander-Universität Erlangen-Nürnberg, 91054 Erlangen, Germany; 3 Clinic and Polyclinic for Internal Medicine II, Klinikum Rechts der Isar, Technische Universität München (TUM), 80333 Munich, Germany; 4 Comprehensive Cancer Center Erlangen-EMN (CCC ER-EMN), 91054 Erlangen, Germany; 5 Department of Gynecology and Obstetrics, University Hospital Erlangen, Friedrich-Alexander-Universität Erlangen-Nürnberg, 91054 Erlangen, Germany; 6 Preclinical Imaging Platform Erlangen (PIPE), Institute of Radiology, University Hospital Erlangen-Nuremberg, 91054 Erlangen, Germany; 7 Experimental Pharmacology & Oncology Berlin-Buch GmbH, 13125 Berlin, Germany

**Keywords:** mouse xenograft, CAM model, MCF-7, MDA-MB-231, HTB-26, tumor aggressiveness, alternative in vivo model, 3R, hormone receptor

## Abstract

**Simple Summary:**

The chorioallantoic membrane (CAM) is a highly vascularized membrane found in avian eggs. Tumor cell lines can be grown on the CAM, which allows for the further analyses of the tumor grafts afterwards. We investigated the biological and growth characteristics of two breast cancer cell lines that resemble two biologically different breast cancer subgroups. Known biological features of the more aggressive breast cancer cell line were clearly confirmed in vitro and in the CAM model. Furthermore, the tissue-based pathological variables assessed in the CAM model were similar to those of the mouse xenografts and human patient tumor tissue. We suggest this in vivo model to be a reliable alternative for breast cancer research to reduce murine animal experiments.

**Abstract:**

The chorioallantoic membrane (CAM) assay is an alternative in vivo model that allows for minimally invasive research of cancer biology. Using the CAM assay, we investigated phenotypical and functional characteristics (tumor grade, mitosis rate, tumor budding, hormone receptor (HR) and HER2 status, Ki-67 proliferation index) of two breast cancer cell lines, MCF-7 and MDA-MB-231, which resemble the HR+ (luminal) and triple-negative breast cancer (TNBC) subgroups, respectively. Moreover, the CAM results were directly compared with murine MCF-7- and MDA-MB-231-derived xenografts and human patient TNBC tissue. Known phenotypical and biological features of the aggressive triple-negative breast cancer cell line (MDA-MB-231) were confirmed in the CAM assay, and mouse xenografts. Furthermore, the histomorphological and immunohistochemical variables assessed in the CAM model were similar to those in human patient tumor tissue. Given the confirmation of the classical biological and growth properties of breast cancer cell lines in the CAM model, we suggest this in vivo model to be a reliable alternative test system for breast cancer research to reduce murine animal experiments.

## 1. Introduction

Invasive breast cancer represents both the most frequently diagnosed type of malignant tumor in women, with 2.25 million new cases worldwide in 2020, and the leading cause of cancer-associated death in women (0.68 million deaths in 2020) [1]. Prognostic and/or predictive clinical and pathological parameters are used for targeted therapy stratification [2]. In the early 2000s, an unsupervised clustering analysis of an mRNA-based gene expression analysis of human breast cancer tissue revealed molecular breast cancer subtypes that have both significant prognostic and predictive relevance. Using a signature of 50 genes (PAM50), invasive breast cancer can be differentiated into four major molecular subgroups: luminal A, luminal B, HER2-enriched, and basal-like [3,4,5,6,7,8]; molecular-like subtyping using widely available and cost-effective immunohistochemistry (and in situ hybridization, if needed) +/− tumor grade is used in daily routine diagnostics to estimate these molecular subtypes. Invasive breast cancer that expresses the two hormone receptors (HRs), estrogen receptor (ER), and progesterone receptor (PR) and presents a negative HER2 status and low expression level of the proliferation marker Ki-67 (+/− low or intermediate tumor grade) is classified as luminal A-like tumors; HR-positive, HER2-negative breast cancer with a high Ki-67 level +/− high tumor grade (G3) is classified as the luminal B-like subtype. Moreover, HER2-positive invasive breast cancer and triple-negative breast cancer (TNBC, i.e., tumors lacking both HR expression and HER2 overexpression/amplification) must be recognized, as they significantly predict prognosis and treatment response. Therefore, the assessment of the molecular(-like) subtypes is mandatory and of high clinical relevance in pathological diagnostics [9,10,11,12,13,14]. Hence, research on breast cancer biology should include the correlation of new findings with molecular(-like) subtypes. In this application, breast cancer cell lines are a valuable and popular option for both in vitro and in vivo studies given their very well characterized biological features, including their molecular(-like) subtype [15,16,17], where they are used, e.g., to obtain more insights into the complexity of breast cancer biology or to test drug susceptibility. For example, while the breast cancer cell line MCF-7 represents an HR+ (luminal) breast cancer subtype, the breast cancer cell line MDA-MB-231 biologically shows triple-negative and more aggressive behavior [18,19].

In the past, in vivo studies that used breast cancer cell lines, including for drug susceptibility testing, were performed using predominantly mouse models [20,21,22,23,24]. Because of the use of this method for many decades, the biology and physiology of many mouse models are very well known, and murine models have been improved to answer specific scientific issues (e.g., humanized mouse models) [25]. Moreover, long observation time [26] and in vivo imaging can be applied to this in vivo model [27]. However, the disadvantages are obvious: Besides the long duration of the experiment, the high costs and large number of animals needed for the mouse model as an animal experiment and the ethical aspects, such as the stress, pain, and suffering of the animals, need to be compared with the scientific gain of knowledge for human cancer patients. Moreover, many organizational aspects, such as the long-term planning phase for the experiments, including the application procedure for animal experiments and necessary Federation of European Laboratory Animal Science Associations (FELASA) courses for the staff, must be considered [28,29,30,31].

In 1959, following the 3R principles (replacement, refinement, and reduction of animals in research), novel recommendations for the use of in vivo experiments have been suggested [32]. The chorioallantoic membrane (CAM) assay is discussed as an alternative in vivo model that allows for tissue transplantation/engineering, minimally invasive research of cancer biology, and the susceptibility of the cancer cells to drugs [33]. Using the CAM model, functional and phenotypical characteristics (e.g., growth and dissemination potential, differentiation of cancers, mitosis and proliferation rates, and expression of clinically relevant prognostic and predictive biomarkers) can be investigated by applying cancer cell lines or patient-derived tumor tissue onto the CAM [34,35,36]. The hallmarks of cancer can be studied in a simplified manner and in a very short time compared with murine models, and because the CAM is not innervated, the inability of the chicken embryo to feel pain is assumed [37,38,39,40,41,42]. For some solid cancer types, i.e., clear cell renal cell cancer (ccRCC), bladder cancer, and prostate cancer, successful engraftment rates of cancer cell lines or patient-derived tumor tissue were reported while using the CAM model. Moreover, for ccRCC, the ovografts showed histopathological and dissemination features similar to those found in ccRCC mouse xenografts [28]. However, to the best of our knowledge, a systematic comparison of the morphological and immunohistochemical features between breast cancer cell line ovografts and mouse xenografts is currently lacking.

Hence, we investigated the phenotypical and functional characteristics (e.g., tumor grade, mitosis rate, tumor budding, HR and HER2 status, and Ki-67 proliferation index) of two breast cancer cell lines with a) different histological features and b) different biological aggressiveness, namely, the HR+ (luminal) cell line MCF-7 and the more aggressive TNBC-like cell line MDA-MB-231, in both the CAM and mouse xenograft models. Moreover, we compared the pathological features of the in vivo xenograft models with those expressed in human primary tumor specimens [43]. Moreover, functional differences in the aggressiveness between both breast cancer cell lines were analyzed in vitro and in vivo using the CAM model.

## 2. Materials and Methods

### 2.1. Cell Lines and Culture Conditions

The human breast cancer cell lines MCF-7 (ATCC^®^ HTB-22^™^) and MDA-MB-231 (ATCC^®^ HTB-26^™^) were obtained from the American Type Culture Collection (ATCC, Manassas, VA, USA). Both cell lines were kept in Dulbecco’s modified Eagle’s medium (DMEM, 41965039, Gibco/Life Technologies, ThermoFisher Scientific, Waltham, MA, USA) supplemented with 10% FBS (P30-3306, PAN Biotech), 1% penicillin/streptomycin (P06-07100, PAN-Biotech GmbH, Aidenbach, Germany), 1% nonessential amino acids (MEM-NEAA, 11140-035, Gibco, ThermoFisher Scientific, Waltham, MA, USA), and 1% sodium pyruvate (S8636, Sigma-Aldrich, St. Louis, MO, USA). Cells were cultured in a humidified atmosphere containing 5% CO_2_ at 37 °C. Cell line verification was performed through a multiplex cell authentication test performed by Multiplexion (Heidelberg, Germany) as described by Castro et al. [44]. Mycoplasma-free status was verified for both cell lines. All cells were used in passages 3 to 10 in the experiments after thawing them from the original stocks. The sources and the clinical and pathological characteristics of the tumors from which the MCF-7 and MDA-MB-231 breast carcinoma cell lines originate are summarized in Table 1 [45,46,47,48].

### 2.2. Human Patient Tissue

To compare the histopathological and immunohistochemical characteristics of the breast cancer cell lines MCF-7 and MDA-MB-231 grown in the chorioallantoic membrane (CAM) xenograft assays, five human hormone receptor-positive, HER2-negative breast cancer samples and five human TNBC samples were used (*n* = 5 each). For each human case, formalin-fixed and paraffin-embedded (FFPE) tumor tissue originating from surgical specimens was available. The hormone receptor-positive, HER2-negative and TNBC status characterization was given in the diagnostic report. However, the histopathological and immunohistochemical (IHC) characterization of the tumors was analogously repeated by a pathologist experienced in breast cancer pathology for the IHC assessment of the breast cancer cell lines (see below) to centrally confirm the hormone receptor-positive, HER2-negative and TNBC status characteristics, respectively. For hormone receptor-positive, HER2-negative breast cancer, the patients’ mean age (all female) at diagnosis was 63.4 years (range of 36 to 82 years). The median tumor size was 2.1 cm (range of 1.9–2.1 cm). One patient presented an invasive lobular breast cancer. For TNBC, the patient’s mean age (all female) at diagnosis was 77.2 years (range of 48 to 92 years). The median tumor size was 11.3 cm (range of 0.6–45.0 cm). One patient presented three foci of TNBC. Two TNBC cases had associated ductal carcinoma in situ (DCIS); one of them had been treated with neoadjuvant systemic therapy without achieving a pathological complete response (pCR; remaining tumor size: 1.6 cm). The youngest patient presented a 45.0 cm tumor. The investigation of human tumor samples was covered by the local ethics committee (ethics vote iMODE-B No. 4514) in accordance with the institutional and national ethical standards on human experimentation and with the Helsinki Declaration. Subjects were properly instructed and provided written consent to participate. Patient privacy and anonymity are fully protected.

### 2.3. Cell Morphology and Fluorescence Staining of Breast Cancer Cell Lines

Representative light microscopy images of the two investigated cell lines MCF-7 and MDA-MB-231 were taken at ~70–80% confluency to compare the cell morphology using a Leica DMi1 light microscope (inverse, 20× objective HI Plan I, Leica Microsystems). Images of three independent cell culture plates per cell line were acquired.

For fluorescence staining, MCF-7 (3 × 10^4^ cells/well) and MDA-MB-231 (2 × 10^4^ cells/well) cells were seeded in ibidi^®^ µ-slide 8-well ibiTreat cell culture plates (80826, ibidi^®^). After 24 h, both cell lines had reached a confluency of ~80% and were fixed with 4% phosphate-buffered formalin for 20 min, permeabilized with 0.1% Triton^TM^ X-100 (T8787, Sigma-Aldrich, St. Louis, MO, USA) solution in PBS (P5368, Sigma-Aldrich) for 15 min, and blocked with 1% bovine serum albumin (BSA, T844.2, Roth) solution in PBS. Then, F-actin was stained with AlexaFluor^®^ 488-conjugated phalloidin (1:80 in PBS, #8878, Cell Signaling), and nuclei were counterstained with Hoechst 33342 (B2261, Sigma-Aldrich). Finally, the cells were covered with PBS solution, and fluorescence images were taken using a Nikon Eclipse Ti-S fluorescence microscope (40× Plan Fluor objective, Nikon).

### 2.4. Spheroid Invasion Assay

To evaluate the invasive potential of the two breast cancer cell lines in vitro, which is predictive of their dissemination potential in vivo, a three-dimensional (3D) spheroid invasion assay was performed [49,50,51,52,53]. In this assay, the tumor cells are embedded in an artificial extracellular matrix (ECM). The degradation and reorganization of the ECM as well as the active migration through the surrounding matrix represent the cellular processes responsible for tumor cell invasion [49,50,51,52,53]. For the spheroid formation, suspensions of MCF-7 and MDA-MB-231 cells were seeded into the wells of a 96-well, round-bottom ultralow attachment (ULA) plate (1 × 10^3^ cells/well in a volume of 200 µL) and incubated under normal cell culture conditions for 72 h to allow for multicellular spheroid formation (one spheroid per well). Then, 100 µL of the medium was carefully removed, and 100 µL of growth factor reduced Matrigel (Corning^®^ Matrigel^®^ Growth Factor Reduced (GFR) Basement Membrane Matrix, phenol red-free, *LDEV-free, 356231, Corning) was added to the remaining 100 µL of the cell culture medium. After mixing the medium and Matrigel by carefully pipetting up and down, the 96-well ULA plate was centrifuged at 300× *g* for 3 min at 4 °C to position the spheroids in the center of each well. Afterwards, the Matrigel was allowed to solidify by incubation under normal cell culture conditions for 1 h. Then, 100 µL of cell culture medium was added on top of the solidified spheroid-containing Matrigel layer, and the initial spheroid size (0 h) was documented using a light microscope (inverse, 4× objective HI Plan I, Leica Microsystems). The invasion area of the breast carcinoma cell lines MCF-7 and MDA-MB-231 was captured after 24 h and 48 h of incubation. The initial size of the spheroids (spheroid area at 0 h) and the invasion area (24 h and 48 h) were measured in the obtained microscopy images using ImageJ software (Version 1.46r, Rasband, W.S., U.S. National Institutes of Health). The relative invasion area was finally determined as a measure of the invasive potential for the investigated cell lines at 24 h and 48 h by dividing the respective invasion area by the initial spheroid size (0 h). The assay was carried out in two independent experiments with a total of 16 replicates per cell line.

### 2.5. Chorioallantoic Membrane Xenograft (CAM Ovograft) Assay

**Figure 1:**
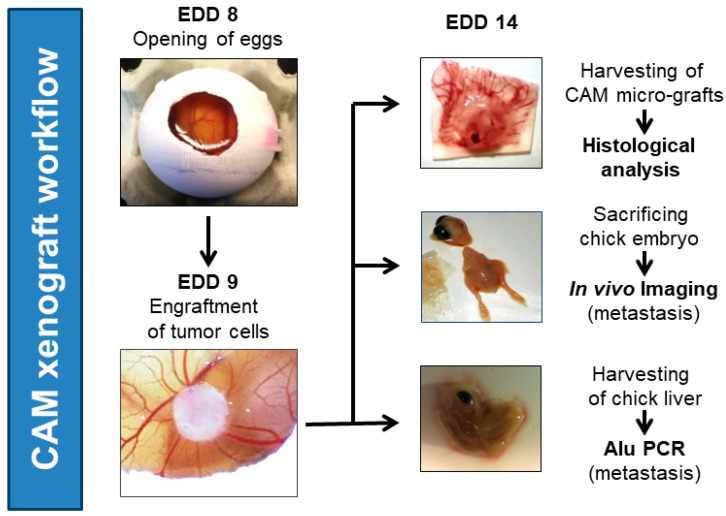
General workflow of in vivo CAM ovograft experiments. Eggs were opened on embryonic development day (EDD) 8. On the next day (EDD 9), tumor cell/Matrigel pellets (1 × 10^6^ tumor cells per pellet; optionally, fluorescence-labeled tumor cells for in vivo imaging) were applied to the CAM. Then, ovografts were allowed to develop and grow for 5 days on the CAM. On EDD 14, CAM ovografts were harvested, embryos were sacrificed by decapitation, and chicken embryo livers were collected for further processing. The downstream investigations included the histological analysis of CAM ovografts, in vivo imaging to identify fluorescently labeled tumor cells in chicken embryos (dissemination), and human-specific Alu qPCR analysis to detect the DNA of human tumor cells in chicken livers (dissemination).

Fertilized specific pathogen-free (SPF) chicken eggs were obtained from Valo Biomedia (Osternholz-Scharmbeck, Germany) and placed in an egg incubator at 37 °C and 70–80% humidity. This day was counted as day 1 because incubation under the aforementioned conditions initiated the embryogenesis. On day 8, a small window was created on the eggshell, and the shell membrane was separated from the CAM by applying a small drop of Dulbecco’s phosphate-buffered saline (DPBS) solution on the former, followed by scarring so that the DPBS flowed between the eggshell membrane and the CAM. This helps to easily remove the shell membrane without disturbing the developing vascular network of the CAM. To prevent drying out and contaminating the CAM, the window was sealed with a piece of silk tape. On day 9, 1 × 10^6^ cells per cell line were transplanted into the CAM, and the eggs were further incubated until day 14. On day 14, the primary tumor was harvested, and the tumor volume was measured as previously described [54] and according to the schematic workflow depicted in Figure 1. Then, the primary tumor was fixed in 4% phosphate buffered formaldehyde for 24 h. As soon as the tumor was harvested, the chick embryos were sacrificed by decapitation. Then, the chicken embryo liver was collected and snap-frozen in liquid nitrogen. Until the DNA isolation for Alu qPCR, the chicken embryo livers were stored at −80 °C.

After harvesting, ovografts were formalin-fixed and embedded into paraffin with *n* = 10 MCF-7 and *n* = 12 MDA-MB-231 ovograft FFPE blocks, respectively, which harbored sufficient tumor tissues for reliable histopathology/IHC assessments. The CAM xenograft experiments were performed according to the Directive 2010/63/EU of the European Parliament and of the Council of 22 September 2010 on the protection of animals used for scientific purposes that do not include any restrictions for the application of nonmammalian embryos [55]. In accordance with the German Animal Experiment and Welfare Guidelines, no ethical approval was needed. A daily check was performed for embryonic viability. The final chicken embryo death ratio was approximately 50% for both MCF7-ovografts and HTB-26 ovografts.

#### 2.5.1. Analysis of the Dissemination Potential In Vivo by Fluorescence Imaging

Analysis of the in vivo dissemination capability of MCF-7 and MDA-MB-231 breast cancer cells was performed in the CAM xenograft assay as described above but with fluorescently prelabeled tumor cells (Cell Proliferation Staining Reagent–Deep Red Fluorescence–Cytopainter, ab176736, Abcam, Cambridge, UK) and as recently reported [54].

#### 2.5.2. Analysis of the Dissemination Potential In Vivo by Human-Specific Alu qPCR

Chicken embryo livers collected at the end of CAM xenograft experiments (EDD 14) were frozen in liquid nitrogen and stored at −80 °C until further use. For dissemination detection using human-specific Alu qPCR, embryonic livers were thawed and dissociated using the gentleMACS^TM^ Octo Dissociator system with M tubes (Miltenyi Biotec) according to the supplier’s instructions. Cell lysis (16 h, 56 °C) and genomic DNA preparation were performed with the NucleoSpin^®^ Tissue kit (Macherey-Nagel) according to the user manual. Quantification and purity of isolated genomic DNA were determined using a Nanodrop^®^ ND-1000 system (Nanodrop). Then, 200 ng of total genomic DNA was amplified by the application of the QuantiTect SYBR^®^ Green PCR kit (Qiagen) and the following primers specific to human Alu sequences (hAlu, Metabion): primer hAlu.sense: 5′-ACGCCTGTAATCCCAGCACTT-3′; and primer hAlu.antisense: 5′-TCGCCCAGGCTGGAGTGCA-3′ [56]. DNA samples were subjected to an initial denaturation at 95 °C for 15 min, followed by 40 cycles at 94 °C for 15 s for further denaturation, 60 °C for 30 s for annealing, and 72 °C for 30 s for elongation. The number of hAlu sequences was normalized to chicken *GAPDH* (chGAPDH), which was detected using the following primers: primer chGAPDH.sense: 5′-GAGGAAAGGTCGCCTGGTGGATCG-3′; and primer chGAPDH.antisense: 5′-GGTGAGGACAAGCAGTGAGGAACG-3′ (Zijlstra et al., 2002). Finally, relative hAlu sequence levels (relative amount of tumor cell dissemination) were determined with respect to a human genomic DNA control sample (human genomic DNA, human mixed, G3041, Promega). The reference human genomic DNA was applied at a concentration of 0.01 ng/mL, and the value obtained for this sample was set to 1.0. The sensitivity limit of the Alu qPCR method could be detected with 0.001 ng/mL of human genomic control DNA, where samples reached a relative value of ~0.3, even though a value of 0.1 would be expected. Therefore, a strict cutoff for tumor cell dissemination detection was defined when using this specific PCR method, and samples with relative values < 0.5 were defined as non-disseminated. The experiment was performed in technical triplicate per sample (MCF-7: *n* = 10, MDA-MB-231: *n* = 12).

### 2.6. Mouse Model (Subcutaneous Murine Xenografts)

A total of 1 × 10^7^ MCF-7 (*n* = 5 animals) and MDA-MB231 cells (*n* = 5 animals) were subcutaneously injected at a cell viability of >95% into 6- to 8-week-old female NMRI nu/nu mice. During the study, all animals received hormonal supplementation with E2D (Sigma-Aldrich, Taufkirchen, Germany). Tumor size was measured using a caliper, and tumor volumes were calculated by using the following equation: tumor volume (TV) = (length × width^2^)/2. The tumor growth of MDA-MB231 tumors was determined for 39 days and initially for 39 days for the MCF-7 tumors. Because no MCF-7 tumors were visible after 39 days, experiments for MCF-7 (*n* = 5 animals) were repeated as described above but for 75 days due to the poor growth potential of the MCF-7 cell line. Then, mice were euthanized under isoflurane anesthesia by cervical dislocation, and tumors were removed for further analyses and documentation. For further histopathological analyses of FFPE murine xenograft tissue, *n* = 2 MCF-7 (due to too small tumor volumes in *n* = 3 samples) and *n* = 4 MDA-MB-231 tumor xenografts (*n* = 1 died on observation day 35) were available. The animal study was conducted at the EPO GmbH Berlin-Buch in compliance with the United Kingdom Coordinated Committee on Cancer Research guidelines and was approved and authorized by the Landesamt für Gesundheit und Soziales, Berlin, Germany (approval No. G 0333/18).

### 2.7. Histopathological Evaluation and Assessment of Immunohistochemical Staining of Breast Cancer Cell Lines Grown in CAM Ovografts, Mouse Xenografts, and Tumor Tissue of TNBC Patients

Serial sections (2 µm) were cut from FFPE blocks of CAM ovografts (both MCF-7 and MDA-MB-231), murine xenografts (both MCF-7 and MDA-MB-231), and human patient tumor tissue and mounted on slides for (immuno-) histopathological evaluation. One section per sample was stained with hematoxylin and eosin (H&E) according to the in-house standard procedures in order to perform the histomorphological analyses. Immunohistochemical (IHC) staining was performed according to the manufacturer’s recommendations and in-house standards (accreditation of the Institute of Pathology of the University Hospital Erlangen according to DIN ISO 17020) on adjacent serial slides using a VENTANA BenchMark ULTRA automatic IHC staining system (Ventana Medical Systems, Tucson, AZ, USA) with the following specific antibodies and dilutions: Phospho-histone H3 (PHH3; monoclonal, 1:200, Biocare Medical, Concord, CA, USA), pancytokeratin AE1/AE3 (polyclonal, 1:40, Zytomed Systems, Berlin, Germany), Ki-67 (monoclonal, clone MIB-1, 1:100 dilution, Dako, Glostrup, Denmark), progesterone receptor (PR) (monoclonal, clone 1E2, ready to use, Ventana Medical Systems), estrogen receptor (ER) (monoclonal, clone EP1; 1:40, Dako), human epidermal growth factor receptor (HER2) (polyclonal, 1:1000, DAKO), androgen receptor (AR) (monoclonal, AR441; 1:50, Dako), cytokeratin 5 (CK5) (monoclonal, clone XM26, 1:50, Zytomed Systems), and synaptophysin (monoclonal, clone Snp88, 1:50, BioGenex, Fremont, CA, USA). Positive and negative controls were performed for each IHC marker. All stained slides were scanned using Pannoramic scanners (3DHISTECH) for digital analyses. For histopathological analyses, Case Viewer software (Version 2.0), Slide Viewer software (Version 2.5, both 3DHISTECH), and a Zeiss Axio microscope (Imager. A2), respectively, were used.

For each tumor, the histological subtype was assessed based on the H&E slides according to the latest *WHO Classification of Tumours: Breast tumours* [57]. The tumor grade was determined according to the grading method described by Elston and Ellis [58].

Both the mitotic count (PHH3 IHC) and tumor budding rate (AE1/AE3 IHC) of tumor cells in the CAM ovografts were determined per high-power field (HPF, 400× magnification); the average number of mitotic figures per HPF and the tumor buds at the tumor invasion front (at least 3 and a maximum of 5 HPFs were evaluated per sample) were determined in each sample. Tumor budding was assessed using pancytokeratin IHC; tumor buds were defined as a single tumor cell or cell cluster of up to 4 tumor cells [59]. The relative vessel density of the CAM ovografts was assessed by an analysis of the H&E-stained tissue sections. For this analysis, the tumor area and then the intratumoral blood vessels were first annotated. The relative vessel density was then calculated by dividing the number of intratumoral blood vessels by the total area of the respective CAM ovografts (number of vessels per mm^2^ of tumor area). Larger intratumoral white spaces resulting from cutting artifacts or larger areas without focus were present in a few scans and were excluded from the analysis [54].

For the assessment of the ER, PR, and AR status, the continuous percentage and intensity of positively stained tumor cells was stated. Moreover, the immunoreactive score (IRS) according to Remmele and Stegner was determined [60]. Positive staining for ER and PR was defined as ≥1% [61]. The IHC expression of the proliferation marker Ki-67 was assessed as a continuous percentage of positively stained tumor cells. The HER2 IHC score was documented as 0, 1+, 2+, or 3+ in accordance with the published guidelines [62]. Tumors with a score of 0 or 1+ were considered HER2-negative, and those with a score of 3+ were defined as HER2-positive. Samples with 2+ staining were analyzed for gene copy numbers of HER2 using the chromogenic in situ hybridization (CISH) method. The *HER2* gene copy numbers (GCN) and centromere GCN of the corresponding chromosome 17 were visualized using a kit with two probes of different colors (ZytoDot^®^ 2C SPEC *ERBB2*/*CEN 17* Probe; Zytomed Systems GmbH, Berlin, Germany). The *HER2* CISH assessment was performed according to the current guidelines [62].

Molecular-like subtyping is explained in Table 2. Briefly, TNBC was characterized by the lack of ER and PR expression and a negative HER2 status. The hormone receptor (HR)-positive, HER2-negative luminal-like tumors were separated into luminal A-like and luminal B-like tumors using tumor grade and/or Ki-67 value. If the tumor had a positive HER2 status, HER2-positive breast cancer was stated; depending on the HR status, it was classified as a luminal B-like HER2-positive BC if HRs were positive, whereas it was classified as HER2-positive (non-luminal) if the expression of HRs was <1%.

For E-cadherin, the given expression was stated as “intact” or the lack of expression, which would serve as an indicator for the histological subtype invasive lobular breast carcinoma, which was stated as “loss”. Furthermore, using CK5, which serves as a marker for basal differentiation, the percentage of positive tumor cells was assessed. For the neuroendocrine marker synaptophysin, the IHC status was evaluated as either negative (no expression) or positive (any expression).

### 2.8. Statistical Analysis

All statistical analyses were performed using GraphPad Prism 7 software (GraphPad Software, Inc., Insight Partners, New York City, NY, USA).

## 3. Results

### 3.1. In Vitro Cell Morphology and Invasive Potential of Breast Cancer Cell Lines

**Figure 2:**
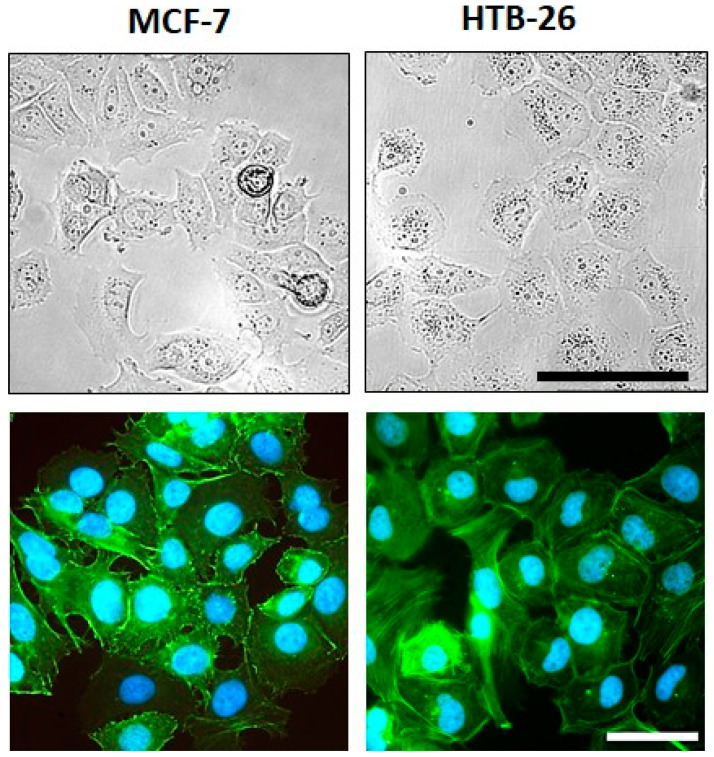
Morphology and F-actin organization of MCF-7 and MDA-MB-231 breast cancer cells. (**Upper row**) Representative images of the cell morphology of MCF-7 and MDA-MB-231 breast cancer cells at ~70–80% confluency in adherent culture; scale bar: 100 µm (applies to both cell lines). (**Lower row**) Examples of fluorescence images of the F-actin organization in MCF-7 and MDA-MB-231 breast carcinoma cells; green—F-actin; blue—nuclei; scale bar: 50 µm (applies to both cell lines).

Both breast cancer cell lines showed viable tumor cells growing in aggregates (Figure 2). The cytoskeleton architecture shown in the immunofluorescent F-actin staining revealed more scattered stress fibers at the borders in MCF-7 cells; in contrast, they were rather well aligned in MDA-MB-231 cells. The nuclear shape was similar between both cell lines, with MDA-MB-231 cells having a slightly more pronounced cytoplasm.

**Figure 3:**
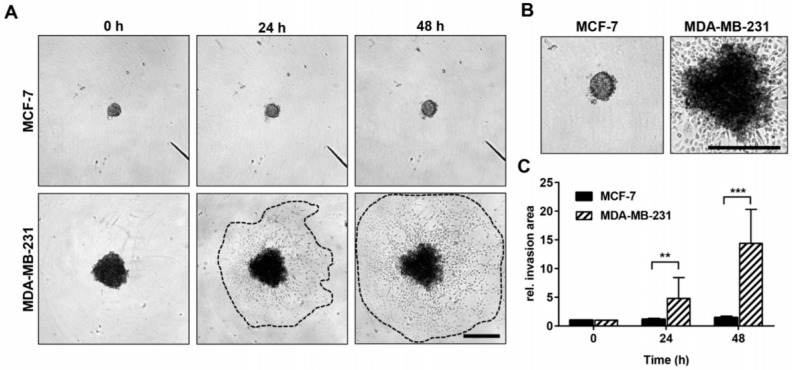
Invasive growth rate of MCF-7 and MDA-MB-231 breast cancer cells in vitro. (**A**) Representative light microscopy images of MCF-7 and MDA-MB-231 spheroids and their surrounding invasion area 0 h, 24 h, and 48 h after embedment into an artificial Matrigel ECM. Dotted lines mark the boundaries of the invasion area of MDA-MB-231 cells. Scale bar: 500 µm (applies to both cell lines and all images). (**B**) Digitally enlarged images of MCF-7 and MDA-MB-231 spheroids already shown in (**A**) after 48 h of incubation. Scale bar: 500 µm (applies to both cell lines and all images). (**C**) Time-dependent invasive growth rate of MCF-7 (*n* = 16) and MDA-MB-231 (*n* = 16) cells (defined as relative invasion area) as determined by dividing the measured invasion area (24 h and 48 h) by the initial spheroid size (area occupied by spheroids at 0 h). Mann-Whitney test: values represent means ± standard deviations (SD). ** indicates *p* < 0.01; *** indicates *p* < 0.001.

**Figure 4:**
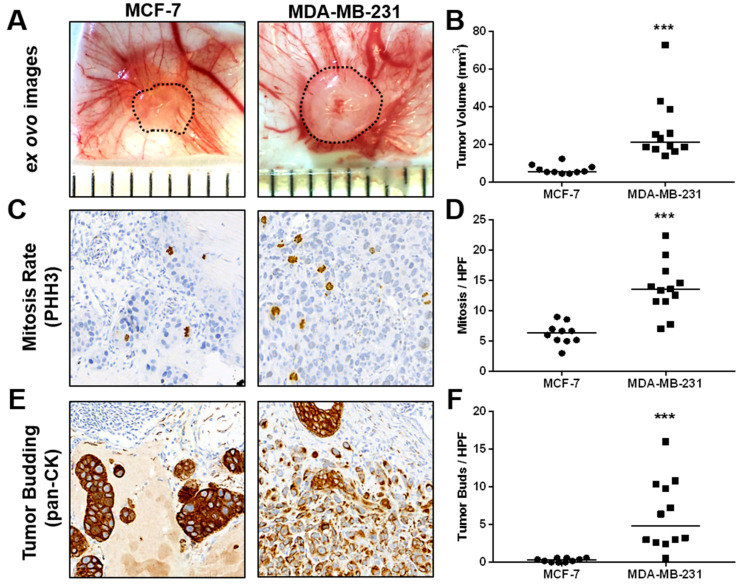
In vivo growth and characterization of tumor aggressiveness of MCF-7 and MDA-MB-231 breast cancer cells in the CAM xenograft assay. (**A**) Example *ex ovo* images of CAM ovografts harvested five days post-engraftment of MCF-7 and MDA-MB-231 cells onto the CAM of fertilized SPF chicken embryos. Scattered lines indicate tumor boundaries. Individual ruler segments define a length of 1 mm. (**B**) Tumor volume of ovografts after 5 days of incubation on the CAM. (**C**) Representative images of mitotic figures as detected in PHH3-stained tissue sections of MCF-7 and MDA-MB-231 CAM ovografts. (**D**) In vivo mitotic rate of MCF-7 and MDA-MB-231 cells as determined by the average number of mitotic figures per high-power field (HPF) in PHH3 IHC-stained tissue sections of CAM ovografts. (**E**) Example images of pancytokeratin-stained tissue sections of MCF-7 and MDA-MB-231 CAM ovografts as applied for the assessment of invasiveness and tumor budding at the tumor invasion front. (**F**) Tumor budding rate of MCF-7 and MDA-MB-231 cells as determined by the average number of tumor buds per HPF at the tumor invasion front in pancytokeratin-stained tissue sections of CAM ovografts. All data are presented with medians, and statistical analyses were performed using the Mann-Whitney test; MCF-7: *n* = 10, MDA-MB-231: *n* = 12; *** indicates *p* < 0.001.

**Figure 5:**
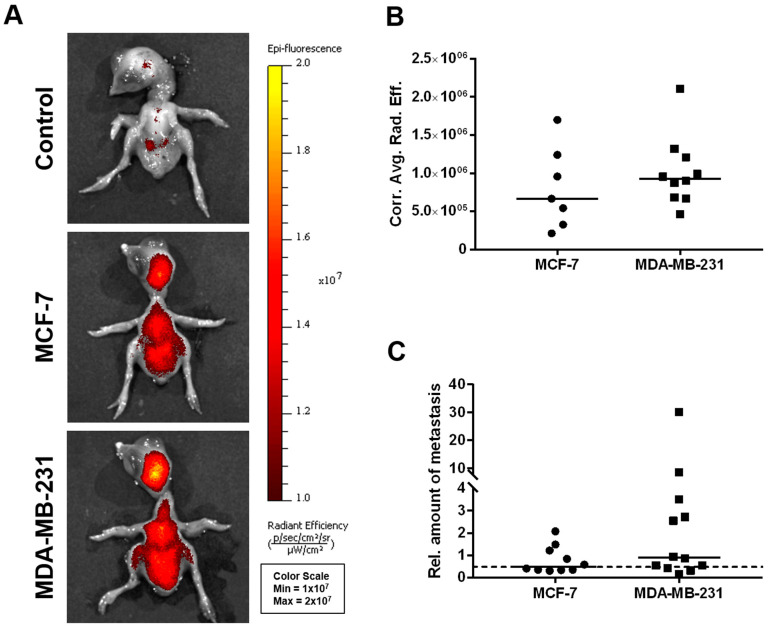
In vivo dissemination capability of MCF-7 and MDA-MB-231 breast cancer cells in the CAM xenograft assay. (**A**) Representative images of chicken embryos as obtained by fluorescence imaging 5 days post-engraftment of deep-red fluorescence-labeled MCF-7 and MDA-MB-231 breast cancer cell pellets onto the CAM (unlabeled cells of both the MCF-7 and MDA-MB-231 cell lines served as controls). (**B**) Corrected average radiant efficiency as assessed by fluorescence imaging in chicken embryos to determine the in vivo dissemination potential of MCF-7 (*n* = 7) and MDA-MB-231 (*n* = 10) cells. (**C**) Relative amount of disseminated tumor cells within the livers of chicken embryos as determined by human-specific Alu qPCR 5 days after engraftment of the breast cancer cell lines MCF-7 (*n* = 10) and MDA-MB-231 (*n* = 12). A value of 1 serves as a reference and indicates an amount of 0.01 ng/mL of human genomic DNA. The dotted line represents the cutoff for tumor cell dissemination detection, which was defined by the sensitivity limit of the Alu qPCR method. Medians of all data are presented as lines in the graphs. For statistical analysis, the Mann-Whitney test was used; the post-analysis was performed using Dunn’s multiple comparison tests.

In the spheroid invasion assay, significant differences were detectable between MCF-7 and MDA-MB-231 cancer cell spheroids. MDA-MB-231 cancer cells invaded significantly deeper into the Matrigel extracellular matrix (ECM) and showed enlarged invasion areas at 24 h and 48 h compared with the MCF-7 cancer cells, which grew only slightly during the observation period (Figure 3). This effect was also observed in vivo (Figure 4 and Figure 5).

### 3.2. In Vivo Growth and Characterization of Tumor Aggressiveness and Dissemination Potential of MCF-7 and MDA-MB-231 Breast Cancer Cells in the CAM Xenograft Assay

Compared with MCF-7 ovografts, MDA-MB-231 CAM ovografts showed significantly larger tumor volumes after harvesting 5 days post-engraftment (Figure 4A,B, Supplemental Figure S1). Furthermore, MDA-MB-231 CAM ovografts presented significantly higher mitotic rates than MCF-7 ovografts (Figure 4C,D). A higher aggressiveness of the MDA-MB-231 cell line was also highlighted by the analysis of tumor budding, where a higher number of tumor buds was detected within the MDA-MB-231 CAM ovografts compared with the MCF-7 ovografts (Figure 4E,F). Although there was a trend, MDA-MB-231 CAM ovografts did not show significantly higher intratumoral vessel density than MCF-7 ovografts. However, their growth appeared to be more aggressive, as shown by the low amount of residual Matrigel compared with MCF-7 ovografts (Supplemental Figure S3A,B).

Using fluorescence imaging of the chicken embryo (Figure 5A,B), no significant differences could be detected between the two cell lines with respect to their dissemination potential; a subset of chicken embryo livers from MDA-MB-231-derived ovografts, however, provided a particularly high signal in the human-specific Alu qPCR analysis (Figure 5C).

### 3.3. Analysis of the Growth Potential of MCF-7 and MDA-MB-231 Breast Cancer Cell Lines in Murine Xenografts

**Figure 6:**
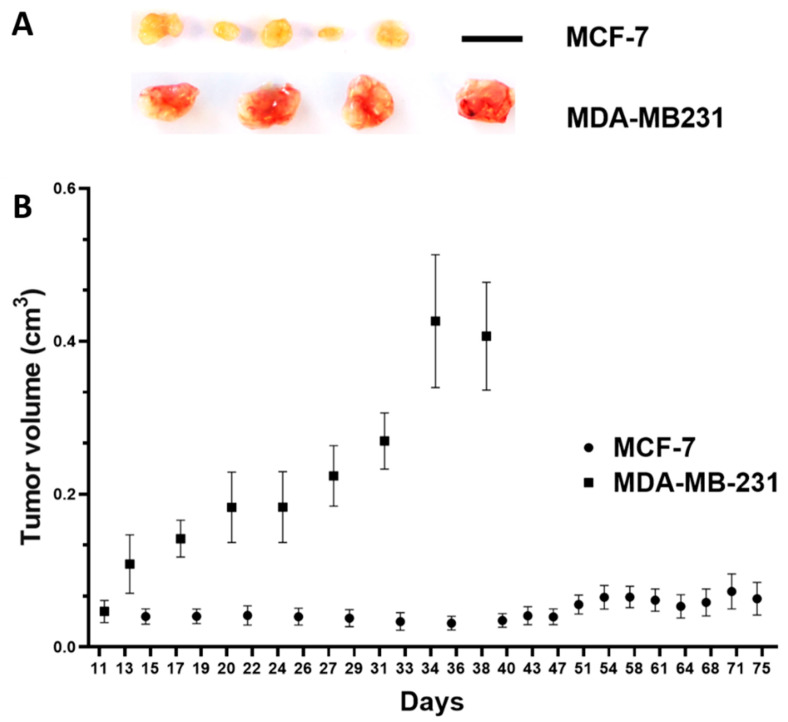
In vivo growth potential of MCF-7 and MDA-MB-231 breast cancer cells in the subcutaneous mouse model. (**A**) Resected tumor xenografts, scale bar: 1 cm (applies to both cell lines). (**B**) Tumor volume in cm^3^ over time (days after inoculation of MCF-7 tumor cells (*n* = 5 animals) and MDA-MB-231 tumor cells (*n* = 4 animals); mean/standard error of the mean).

Investigating the growth potential, the MCF-7-derived xenografts in the cutaneous mouse model were significantly smaller, which showed their lower tumor-forming capabilities (Figure 6A,B).

### 3.4. Histological Comparison of MCF-7 and MDA-MB-231 CAM Ovografts vs. Mouse Xenografts vs. Human Patient Tumor Tissue

**Figure 7:**
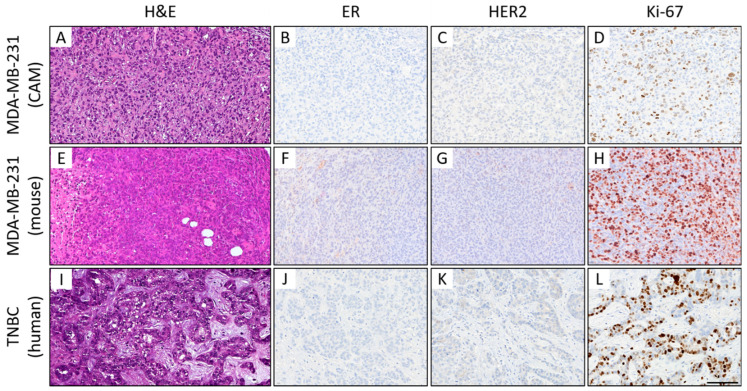
Histological (**A**–**C**) and immunohistochemical comparison of MDA-MB-231 CAM ovografts (**A**–**D**), MDA-MB-231 murine xenografts (**E**–**H**), and human TNBC tumor tissue (**I**–**L**). Representative images of the histomorphology (hematoxylin and eosin) and immunohistochemical expression profile: Expression of estrogen receptor (ER; **B**,**F**,**J**), human epidermal growth factor receptor 2 (HER2; **C**,**G**,**K**), and the proliferation marker Ki-67 (**D**,**H**,**L**) (magnification: x400 each). All three tumors presented a TNBC phenotype with a high-grade morphology, negativity for ER/PR (PR not shown) and HER2, and a high Ki-67 proliferation index. Of note, the mouse xenografts showed some nonspecific background staining (for higher magnification, see Supplemental Figure S4), which should not be confused with specific nuclear and membranous staining of the tumor cells. Scale bar: 100 µm (applies to both cell lines and all images).

**Figure 8:**
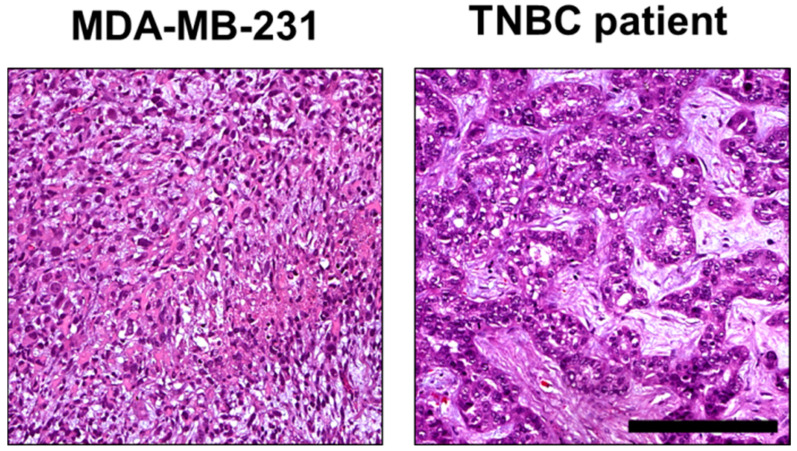
Comparison of histomorphology in MDA-MD-231 CAM ovografts and TNBC patient tumor tissue. Higher magnification of the MDA-MB-231 ovograft (Figure 7A) and human TNBC case (Figure 7I). These images display tumor growth patterns similar to those observed in tumor areas of both MDA-MB-231 CAM ovografts and TNBC patient tumor tissue. In both cases, the TNBC tumor cells showed high pleomorphism and partial cord-like growth surrounded by fibromyxoid tumor stroma (hematoxylin and eosin; each at 200× magnification). Scale bar: 200 µm (applies to both cell lines).

Histomorphologically, MCF-7 and MDA-MB-231 cell lines were presented in both the CAM ovograft and murine xenograft as an invasive breast cancer of no special subtype (NST). Of note, some of the MDA-MB-231 ovografts appeared to grow with looser cell cohesion, which may be due to expansion within the Matrigel; the classical pattern of invasive lobular carcinoma was not observed. For MDA-MB-231, the tumor grade was G3 in both the CAM ovografts and murine xenografts. Immunohistochemistry analyses confirmed the negative status for ER, PR, and HER2 in MDA-MB-231 cells in both of the in vivo models. For the MDA-MB-231 cells, the Ki-67 proliferation index was high (median in the CAM ovograft: 30.0%; median in mouse xenograft: 52.5%; Figure 7A–H). The human TNBC controls consisted of five invasive breast cancer samples (NST), each with poor differentiation (G3). Here, the molecular-like subtype was TNBC (ER/PR/HER2 each negative) for each sample. The median Ki-67 expression was 65.0% (Figure 7I–L). The comparison of MDA-MB-231 ovografts, MDA-MB-231 murine xenografts, and human TNBC tissue showed similar tumor growth patterns and cytology. Figure 8 shows the tumor cells of the MDA-MB-231 ovografts and a human TNBC presenting high pleomorphism and a partly cord-like growth surrounded by fibromyxoid tumor stroma.

**Figure 9:**
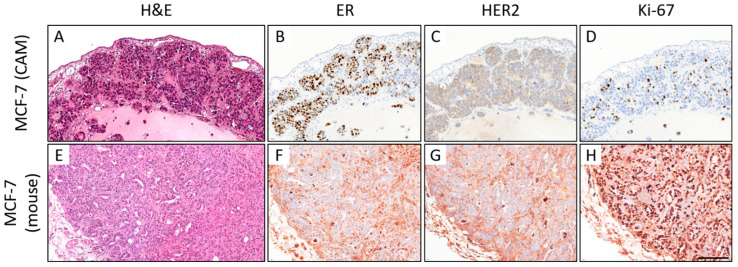
Comparison of the histomorphology (hematoxylin and eosin; H&E) and immunohistochemical expression of estrogen receptor (ER), HER2, and Ki-67 in MCF-7 CAM ovografts (**A**–**D**) vs. MCF-7 mouse xenografts ((**E**–**H**); 400x magnification each). In the CAM ovograft, rather solid growth can be observed, whereas some tubular formations can be detected in the mouse xenograft (**A**,**E**). ER expression can be detected in both grafts (**B**,**F**). Neither the ovograft nor the mouse xenograft showed HER2 overexpression. However, the Ki-67 proliferation index was higher in the mouse xenograft, which was harvested on day 76 (CAM: ovograft harvested 5 days after engraftment). Note that there is significant nonspecific background staining in the immunohistochemical slides of the mouse xenograft, which hampers the biomarker assessment (**F**–**H**). This should not be confused with a specific staining of the tumor cells (for higher magnification, see Supplemental Figure S5). Scale bar: 100 µm (applies to both cell lines and all pictures).

In the MCF-7 ovografts, most tumors had a poor tumor grade (G3, *n* = 7), and the remaining cases were of intermediate grade (G2, *n* = 3). Moreover, MCF-7 ovografts showed luminal A-like (*n* = 3) and luminal B-like (HER2-negative) subtypes (*n* = 7) (ER/PR-positive, HER2-negative; Figure 9). The occurrence of both luminal subtypes was due to the varying tumor grade and Ki-67 proliferation index (median: 15.0%). The murine MCF-7 xenografts (*n* = 2) showed an intermediate (G2) and poor tumor grade (G3) and a luminal B-like phenotype with ER/PR positivity, negative HER2 status, and a median Ki-67 of 75.0%. However, the immunohistochemical assessment was hampered due to the unspecific background in the mouse xenografts (Figure 9E–H). When analyzing the human control cases, a hormone receptor-positive, HER2-negative status was confirmed by our central tumor grade and IHC; two cases presented luminal A-like tumors, and three presented luminal B-like breast cancer. Four cases were classified as invasive breast cancer NST, and one case was of the invasive lobular histological subtype with E-cadherin loss. The tumor grade was G2 in four cases and G3 in one sample. The median Ki-67 was 20.0%, which was close to the median proliferation rate of the MCF-7 ovografts.

With respect to the IHC markers AR (IRS 0/12-2/12), CK5 (mostly negative), and synaptophysin (negative), there were no significant differences between both cell lines and both in vivo models. Table 3 compares the most important immunohistochemical analyses of the MDA-MB-231 and MCF-7 CAM ovografts with MDA-MB-231/MCF-7 mouse xenografts and human hormone receptor-positive, HER2-negative and TNBC tumor tissue, showing good comparability between the in vivo models.

## 4. Discussion

In the present study, we investigated the phenotype and biological behavior of an aggressive, triple-negative breast cancer cell line (MDA-MB-231) and a luminal breast cancer cell line (MCF-7) both in vitro and in vivo using the CAM model. The histopathological features observed in the CAM ovografts were directly compared with those of murine xenografts and human breast cancer tissue. We investigated different hallmarks of cancer, e.g., proliferation, invasion, dissemination, and angiogenesis [63,64], as well as components of molecular-like subtyping, which is a prognostic and predictive biomarker that is mandatory in routine clinical diagnostics.

### 4.1. Investigating the Hallmarks of Cancer Using the CAM Model

Using the CAM assay, the TNBC-like MDA-MB-231 cell line showed a higher growth and invasion potential and a higher mitotic rate than the MCF-7 cells, which is in line with our findings and other in vitro findings [45,65].

Dissemination Capability and Tumor Budding

Moreover, a higher dissemination capability of MDA-MB-231 cells in the ovografts, which was assessed by the analysis of tumor budding, was observed in our study. This pathologically assessed biomarker is defined as small clusters of cancer cells (<5 cells) that are detached from the main tumor mass. Cells in these buds are proposed to actively disseminate from the primary tumor in the first step of dissemination. Hence, in a variety of cancers, including breast cancer, tumor budding has been shown to be a prognostic indicator [59,66,67,68,69]. For human breast cancer tissue, it has been shown that the assessment of tumor budding can be easily implemented in the histopathological biomarker assessment of breast cancer [67]. For breast cancer cell lines, however, little is known about tumor budding in the literature. In contrast, our group had already demonstrated the feasibility of tumor budding assessment in the CAM model using human colorectal cancer cell lines and corresponding knockout cell lines [70].

Although a subset of chicken embryo livers from MDA-MB-231-derived ovografts showed a particularly high Alu qPCR signal, there was no significant difference in regard to the dissemination capability of MDA-MB-231 compared with MCF-7 when using fluorescence imaging. The lack of a significant difference is in line with other findings. Although it is assumed to be highly invasive in vitro, the MDA-MB-231 cell line has been presented as rather poorly disseminating in in vivo models [16]. Although fluorescence imaging allows for the detection of overall dissemination and patterns, Alu qPCR is superior when small differences have to be detected [71,72].

### 4.2. Findings on Histopathological and Molecular Prognostic and Predictive Breast Cancer Biomarkers Using Ovografts

In our study, both the histological subtype (NST) and tumor grade (G3; including solid growth pattern, high nuclear pleomorphism, and high mitotic rate), clinically relevant prognostic and predictive biomarkers [58,73,74], were comparable for MDA-MB-231 cells in both in vivo models (CAM vs. mouse) and in human patient TNBC tissue. In the work of Mota et al., who investigated the IHC expression of ER, PR, HER2, Ki-67, and CK5, MDA-MB-231 cells presented a triple-negative status (although an expression of <10% for ER/PR/HER2 is shown in Figure 3 of their publication; details on the exact percentages are not shown) [43]. In line with these findings, we confirmed the negative ER, PR, and HER2 status for both of the MDA-MB-231 in vivo models (CAM and mouse), hence matching the known phenotype of this breast cancer cell line. The unspecific background of IHC staining due to the cross-reactivity of antibodies was more frequent in the murine xenografts. This issue hampered IHC assessments (ER, PR, HER2, Ki-67) and led to the exclusion of the tumor budding (pancytokeratin to be used) and PHH3 IHC assessments, respectively. Nevertheless, for both MCF-7 and MDA-MB-231, the IHC results of the ovografts and murine xenografts were comparable, which showed a luminal breast cancer subtype (ER-positive, HER2-negative) and triple-negative subtype, respectively.

Proliferation Marker Ki-67 and Mitosis Marker PHH3

Regarding Ki-67, Mota and colleagues reported a Ki-67 proliferation index of > 80% for both the MCF-7 and MDA-MB-231 breast cancer cell lines using paraffin-embedded cell pellets; this is shown in Figure 3 of their publication. However, the details on the exact Ki-67 percentages are not shown [43]. In another breast cancer cell line characterization study, high Ki-67 levels in cell line pellets were confirmed for both the MCF-7 and MDA-MB-231 cell lines (90.0% and 100.0%, respectively) [67]. A possible reason could be that cell pellets do not represent the complex 3D growth of xenografts that also have contact with the stroma and ECM of the host. Of further note, MCF-7 was defined as a luminal A cell line; they did not use the Ki-67 expression or tumor grade for the classification but only used the ER and/or PR positivity and negative HER2 status [67]. In our study, the median Ki-67 proliferation index of MDA-MB-231 was the lowest in our CAM ovografts (30.0%) compared with the murine xenografts (52.5%) and human TNBC tissues (62.5%). Nevertheless, all values reflected a highly proliferative growth.

In human breast cancer diagnostics, the optimal use of Ki-67 is still under debate due to the lack of international standardized protocols; however, efforts have already been made to solve this issue [75]. In our study, we cannot directly compare our in vivo models regarding the Ki-67 values due to the varying observation periods and different tumor microenvironments. In the mouse model, animals with MDA-MB-231 xenografts were observed for 39 days (median Ki-67 of 52.5%) and those with MCF-7 xenografts for 75 days (median Ki-67 of 75.0%); ovografts in general were only harvested 5 days after tumor cell engraftment, which might explain the lower ovograft Ki-67 values (MCF-7: median Ki-67 of 15.0%; MDA-MB-231: 30.0%) compared with those seen in the murine xenografts. Interestingly, the less aggressive cell line MCF-7 was presented as a murine xenograft with the highest Ki-67 levels after the longest observation period, which was 15 times higher than the observation period in the CAM assay. Moreover, the first experimental setup for the MCF-7 mouse experiments passed without results. Therefore, we can note two points of discussion here. First, Ki-67 might not be a perfect marker for comparing the superiority of one of the two in vivo models due to the varying observation periods. However, in the CAM model, MCF-7 ovografts presented lower Ki-67 than the MDA-MB-231 ovograft, indicating a less aggressive biological behavior of the luminal breast cancer cell line compared with the triple-negative one, which was in line with our expectations. In contrast, a study investigating the features of breast cancer cell line-derived spheroid xenografts in athymic nude rats reported both higher Ki-67 levels and PHH3 counts in MCF-7 xenografts than in MDA-MB-231 xenografts [76]. Nevertheless, the mitosis marker PHH3 might be a good alternative for Ki-67 [77], but this marker is not used in routine breast cancer diagnostics.

In our CAM assay, the mitotic rate significantly differed between both breast cancer cell lines when using the PHH3 IHC method, with more frequent mitoses being featured in the aggressive triple-negative cell line. Unfortunately, due to the nonspecific staining background in the murine xenografts, a reliable assessment was not possible for those xenografts. Hence, we could not compare the PHH3 in the ovografts with the PHH3 in the murine xenografts. Second, we can state that the experimental time can be significantly reduced when using the CAM assay (overall, 14 days per egg during embryonic development).

Moreover, there was no unexpected need to repeat experiments for this in vivo model, which contrasts with the long-lasting mouse experiments in which we had to perform the experiments for the very slowly growing MCF-7 breast cancer cell line twice (i.e., first experiments in 6–8-week-old mice + 39 days of observation + second experiments in 6–8-week-old mice + 75 days of observation). Even then, the mean tumor volume of MCF-7 murine xenografts was only 1/10th of that of the MDA-MB-231 murine xenografts, and therefore, it was very difficult to obtain sufficient material for further histopathological analyses (*n* = 2/10). The varying growth profile of MCF-7 cells in the two different in vivo models is quite intriguing. However, in mice, the site of implantation and specific mouse strains affect actual tumor growth. In this study, we used s.c. tumor implantation in NMRI nu/nu mice for both tumor cell lines to better compare the tumor growth and related parameters.

### 4.3. Advantages of the CAM Model

Following the 3R principles (replacement, refinement, and reduction of animals in research) of Russel and Burch as published in 1959, animal experiments should be replaced by alternatives, reduced in regard to the experiment number, and, if not replaceable, models should be modified in a way such that animal suffering is limited to the lowest level [32]. The CAM in vivo model is less invasive than mouse models. This enables the analysis of tissue grafts, the growth and dissemination potential of neoplasms, and angiogenic and antiangiogenic molecules.

Moreover, it is a relatively easy to handle, highly reproducible, reliable, time-effective, and low-cost model that allows for screening experiments and high throughput analyses in a short experimental time. Within 5 days, tumor sizes of up to 1 cm may be seen with a reasonable quantity of tumor cells engrafted, which allows for ovograft growth observation using the naked eye of the investigator. However, one must admit that reaching a sufficient tumor size for further tissue analyses may be hampered if engrafted tumor cells are only slowly growing. Animal protocol approvals are not required for the use of the CAM model (country-dependent). Moreover, being naturally immunodeficient until embryonic day (ED) 10, transplantations from different tissues and species may be performed with only minor immune responses, whereas at ED16, cytotoxic T cells and monocyte populations are available [78]. Furthermore, in contrast to the CAM model, murine in vivo models allow for a longer observation time. Additionally, detecting metastasis in the chicken embryo might be challenging due to the short time period of growth and a circulation pattern that differs from the human pattern. However, for both in vivo models (CAM and mouse), the biological and physiological settings are well discovered. Moreover, both allow for in vivo imaging (summarized in [28,33,36,38,78,79,80]).

Additionally, one has to account for a) the time required for preparing and approving the animal testing application and b) the acquisition, housing, and care of the mice, which is associated with significantly higher costs [28]. In regard to the IHC staining, the murine xenografts more frequently showed an unspecific background staining, which hampered IHC assessments due to cross-reactivities. In contrast, all IHC staining except for one sample could be easily and reliably assessed in the ovografts when using our standardized IHC protocols used for our human pathology routine diagnostics. This, again, saves time and costs by avoiding the establishment of modified IHC protocols.

To date, comparisons between different xenograft models are lacking in the literature. However, there are few studies for the biomarker assessment in glioblastomas [81] and human epidermoid carcinoma cell line (HEp3) [82,83] and for the early evaluation of cancer therapy and early drug toxicity [84], which already shows that the CAM model is an excellent alternative for in vivo studies.

There are several studies about breast cancer using the CAM assay approach; the anticancer drug effects of phospholipase D2 (PLD2) and histone deacetylase (HDAC) inhibitors of the palladium complex BTC2 have been shown [85]. A novel hepatic micrometastatic model of TNBC with 3D tissue engineering constructs and decellularized chick embryo liver scaffolds might be a suitable platform for investigating the mechanisms of organotropism [86]. Similarly, a comparative study between MDA-MB-231 cells and brain-tropic MDA-MB-231 cells allowed us to draw conclusions about their stem cell activity and tumorigenic capacity [87]. In this regard, a CAM-LDA (limiting dilution assay) method has been developed as a tool to determine the stem cell activity of breast cancer cells [88].

However, more direct comparisons including CAM and mouse xenografts are necessary to finally provide convincing argumentation to replace harmful animal experiments.

## 5. Conclusions

In conclusion, we successfully demonstrated the feasibility of using the in vivo CAM assay to show differences in the phenotypical and biological features of the aggressive triple-negative breast cancer cell line MDA-MB-231 and less aggressive luminal breast cancer cell line MCF-7. All histopathological/immunohistochemical biomarkers recommended for diagnosing early invasive breast carcinoma using the German national S3 guideline [14] were similar in the ovografts, murine xenografts, and human patient tumor tissue controls. Thus, we strongly suggest that before designing an in vivo experiment, researchers should consider whether the CAM model could be used instead of rodent models.

## Figures and Tables

**Table 1:** Characteristics of the human breast carcinoma cell lines MCF-7 and MDA-MB-231.

Cell Line	ER	PR	HER-2	Gene Cluster	Tumor Type	Source	Patient
**MCF-7**	+	[+]	–	Luminal	Mammary adenocarcinoma/invasive ductal carcinoma	Metastatic site (pleural effusion)	69-year-old Caucasian
**MDA-MB-231**	–	[–]	–	Basal-like *	Malignant, poorly differentiated epithelial tumor of the breast/adenocarcinoma	Metastatic site (pleural effusion)	51-year-old Caucasian

Source and clinical and pathological characteristics of the tumors are given. ER—estrogen receptor; PR—progesterone receptor; HER-2—human epidermal growth factor receptor 2. (+) indicates expression, and (–) indicates no expression; square brackets indicate that values were derived from mRNA values alone when protein data were not available in the original publication. * Corresponding molecular-like subtype: triple-negative breast cancer (TNBC) [45,46,47,48].

**Table 2:** Molecular-like subtyping of invasive breast cancer using ER, PR, HER2, and Ki-67 expression and tumor grade [9,10].

Molecular-like Subtype	Subgroup	ER	PR	HER2		Grading		Ki-67 (%)
**Luminal A-like**		+	+/−	-	and	G1, G2	or	Low (<20%)
**Luminal B-like**	**HER2-negative**	+	+/−	-	and	G3	or	High (≥20%)
	**HER2-positive**	+	+/−	+		G1, G2, G3		Any value
**HER2-positive (nonluminal)**		-	-	+		G1, G2, G3		Any value
**Triple-negative**		-	-	-		G1, G2, G3		Any value

**Table 3:** Comparison of MCF-7 and MDA-MB-231 CAM ovografts with MCF-7 and MDA-MB-231 mouse xenografts and human TNBC tumor tissue. The results obtained from the immunohistochemical evaluation of the in vivo CAM ovografts in comparison to tissue sections of MCF-7 and MDA-MB-231 murine xenografts and human hormone receptor-positive, HER2-negative breast cancer and TNBC FFPE samples showed that the CAM assay is a good alternative in vivo model for studying breast cancer phenotypes. Proliferation was determined as the percentage of proliferating tumor cells as assessed by Ki-67 staining. (+) indicates IHC positivity, (–) indicates IHC negativity. ER—estrogen receptor; PR—progesterone receptor; HER2—human epidermal growth factor receptor 2. MCF-7 CAM: *n* = 10; MDA-MB-231 CAM: *n* = 12; MCF-7 mouse: *n* = 2; MDA-MB-231 mouse: *n* = 4; hormone receptor-positive, HER2-negative breast cancer patients: *n* = 5; TNBC patients: *n* = 5. An histological comparing of both MCF-7 CAM and MDA-MB-231 CAM ovografts is given in Supplemental Figure S2.

Xenograft/Tumor	ER	PR	HER2	Proliferation (Ki-67)
**MCF-7 CAM ovograft**	+	+	–	Low Median: 15.0%
**MDA-MB-231 CAM ovograft**	–	–	–	High Median: 30.0%
**MCF-7 mouse xenograft**	+	+	–	High Median: 75.0%
**MDA-MB-231 mouse xenograft**	–	–	–	High Median: 52.5%
**Human hormone receptor** **-** **positive, HER2** **-** **negative tumor tissue**	+	+	–	Intermediate Median: 20.0%
**Human TNBC tumor tissue**	–	–	–	High Median: 65.0%

## Data Availability

Data not reported here are available upon request if the request is of an academic/scientific nature.

## Supplementary Materials

The following supporting information can be downloaded at https://www.mdpi.com/article/10.3390/cancers15061704/s1: Figure S1: Positive and negative controls of IHC staining; Figure S2: Comparison of MCF-7 CAM ovograft vs. MDA-MB-231 CAM ovograft; Figure S3: Intratumoral vessels within an MCF-7 ovograft; Figure S4: Higher magnification of nonspecific background staining of estrogen receptor (ER) and HER2 IHC in MDA-MB-231 mouse xenografts; Figure S5: Higher magnification of nonspecific background staining of estrogen receptor (ER) and HER2 IHC in MCF-7 mouse xenografts.
