# Supplementary material for: The Chorioallantoic Membrane Xenograft Assay as a Reliable Model for Investigating the Biology of Breast Cancer
Source: Cancers (Basel). 2023 Mar 10;15(6):1704. doi: 10.3390/cancers15061704 (PMC10046776; doi:10.3390/cancers15061704)
Supplement: Supplementary file 1 [file cancers-15-01704-s001.zip › cancers-2226388-supplementary.pdf]

Supplemental Figure S1

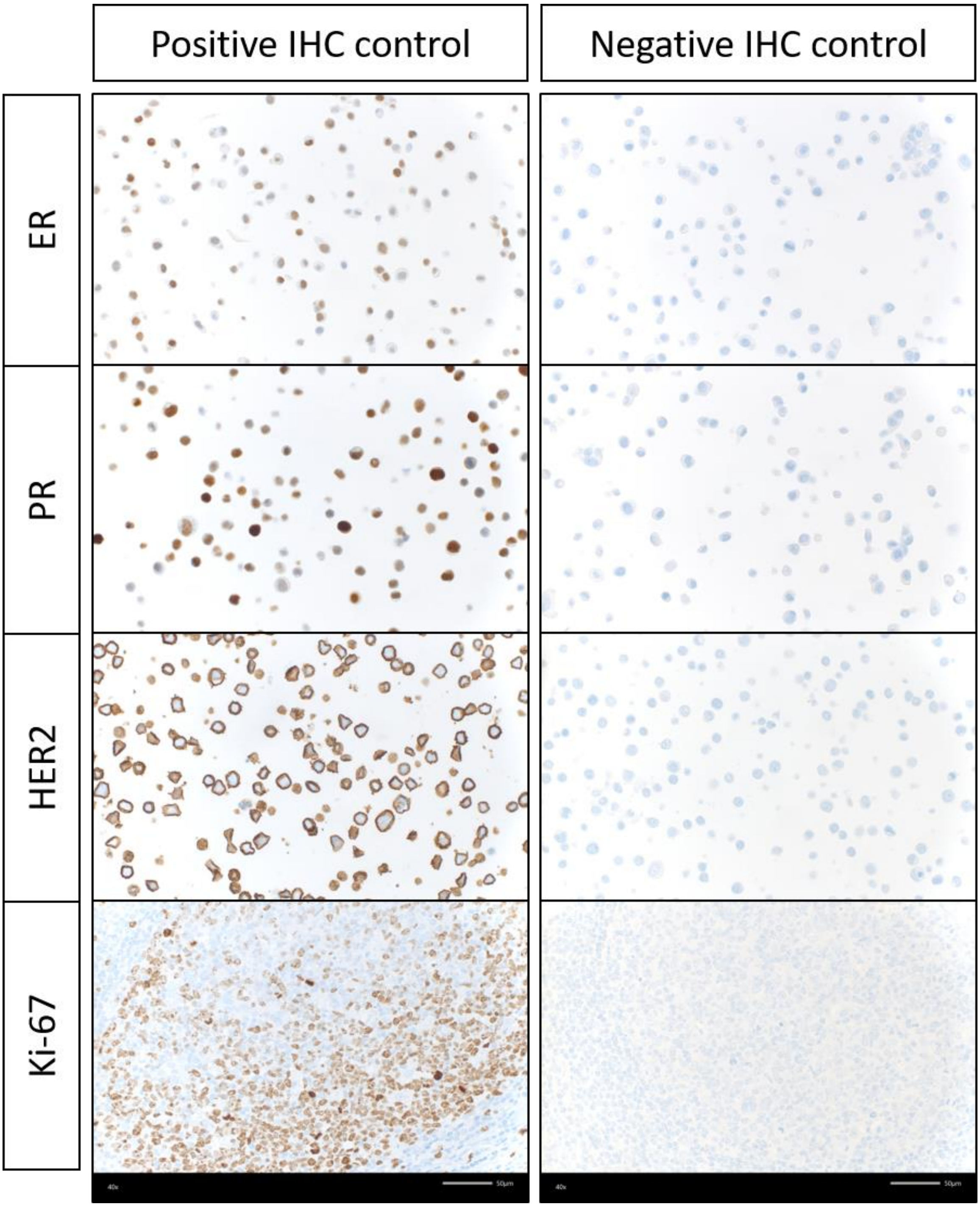

Figure S1: Positive and negative controls of IHC staining. For each IHC staining, positive and negative controls were used to confirm successful specific IHC staining procedures. For both positive and negative IHC controls, the control tissue was as follows: marker-specific breast cancer cell lines for estrogen receptor (ER), progesterone receptor (PR), and HER2; lymph node for Ki-67 and PHH3; epidermis for pancytokeratin, CK5, and E-cadherin; and colon for synaptophysin. To obtain the negative controls, the primary antibody was omitted. The left column shows the positive control for estrogen receptor (ER), progesterone receptor (PR), HER2, and Ki-67 IHC, and the right column shows the negative control for those markers (each, x400 magnification). Scale bar: 50 µm.

Supplemental Figure S2

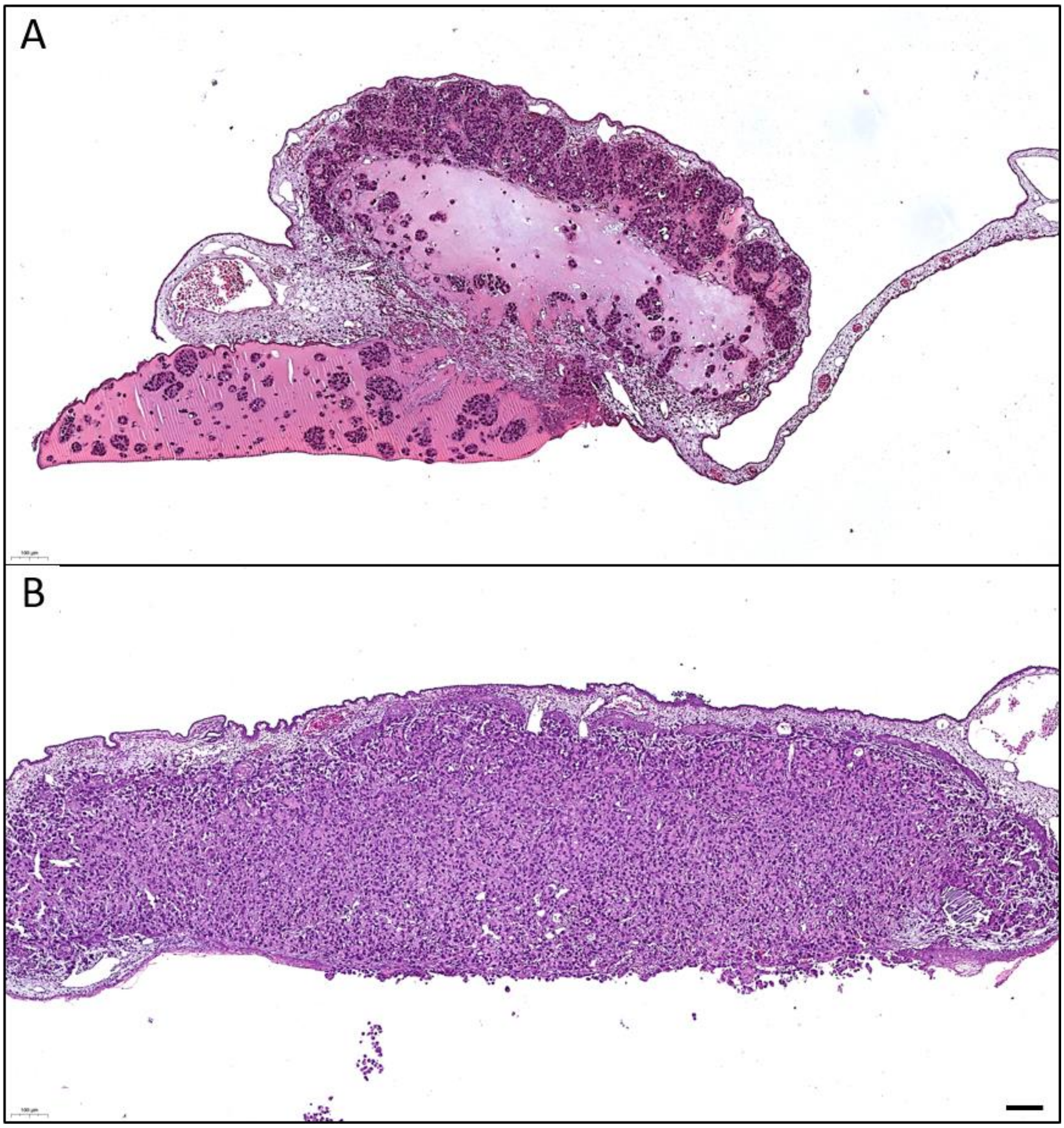

Figure S2: Comparison of MCF-7 CAM ovograft (A) vs. MDA-MB-231 CAM ovograft (B) shows a significantly larger tumor area/tumor size of the TNBC cell line MDA-MB-231 (each hematoxylin & eosin staining, x100 magnification). Scale bar: 100 µm *(*applies to both cell lines*)*.

Supplemental Figure S3

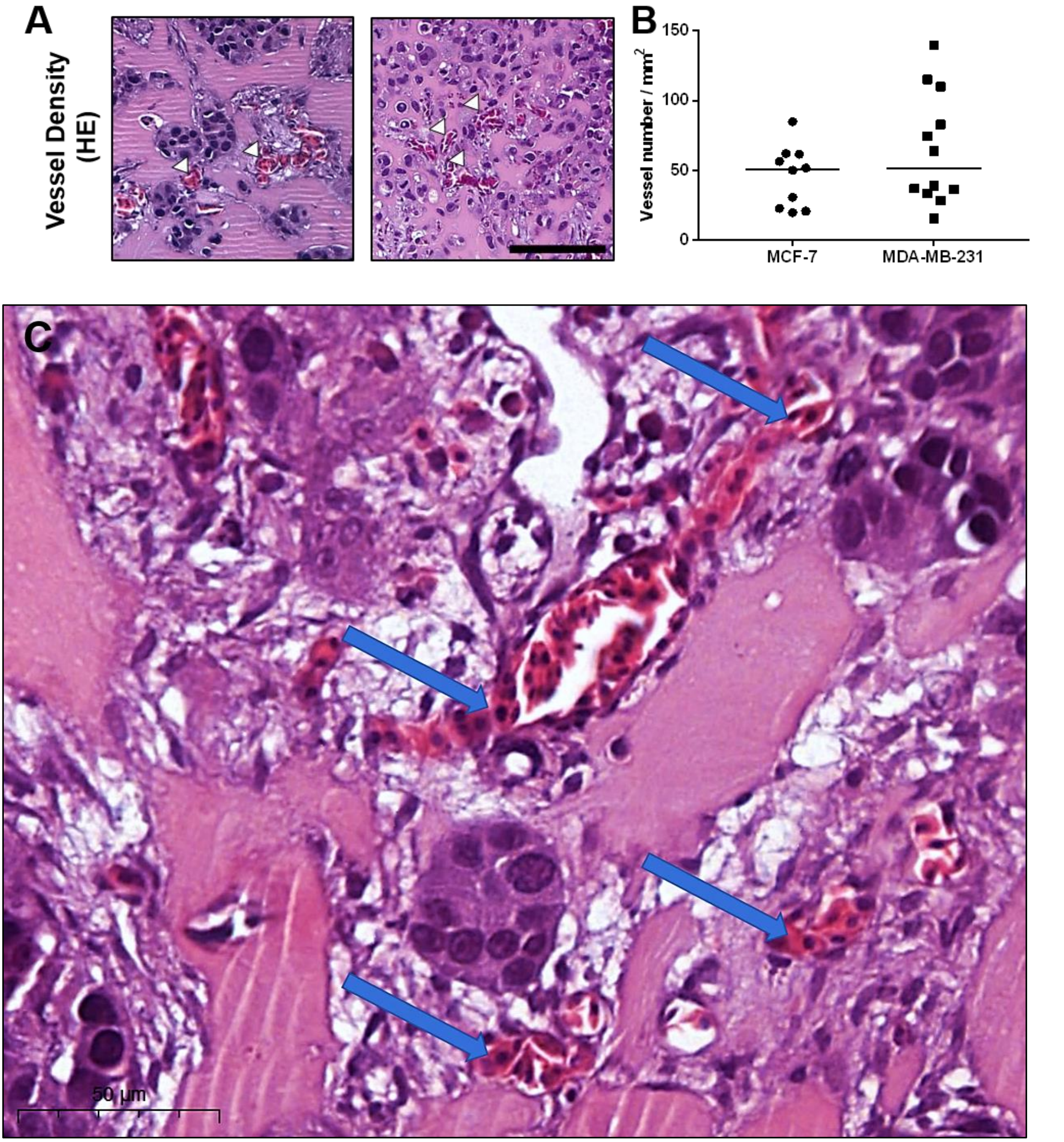

Figure S3: (A) Representative images of H&E-stained tissue sections of MCF-7 and MDA-MB-231 CAM ovografts as applied for the analysis of the intratumoral vessel density (arrowheads highlight vessels). (B) Relative vessel density of MCF-7 and MDA-MB-231 CAM ovografts as determined by the number of vessels per mm2 of tumor mass in H&E-stained tissue sections. Vessels can be easily detected due to the intravasal nucleated erythrocytes (arrows highlight some of the vascular structures; a higher magnification of intravasal nucleated erythrocytes is given (C). (C) Higher magnification of vessels within an MCF-7 ovograft. Vessels can be easily identified due to the intravasal nucleated erythrocytes highlighted by arrows (hematoxylin & eosin staining, x1000 magnification). Scale bar: 50 µm.

Supplemental Figure S4

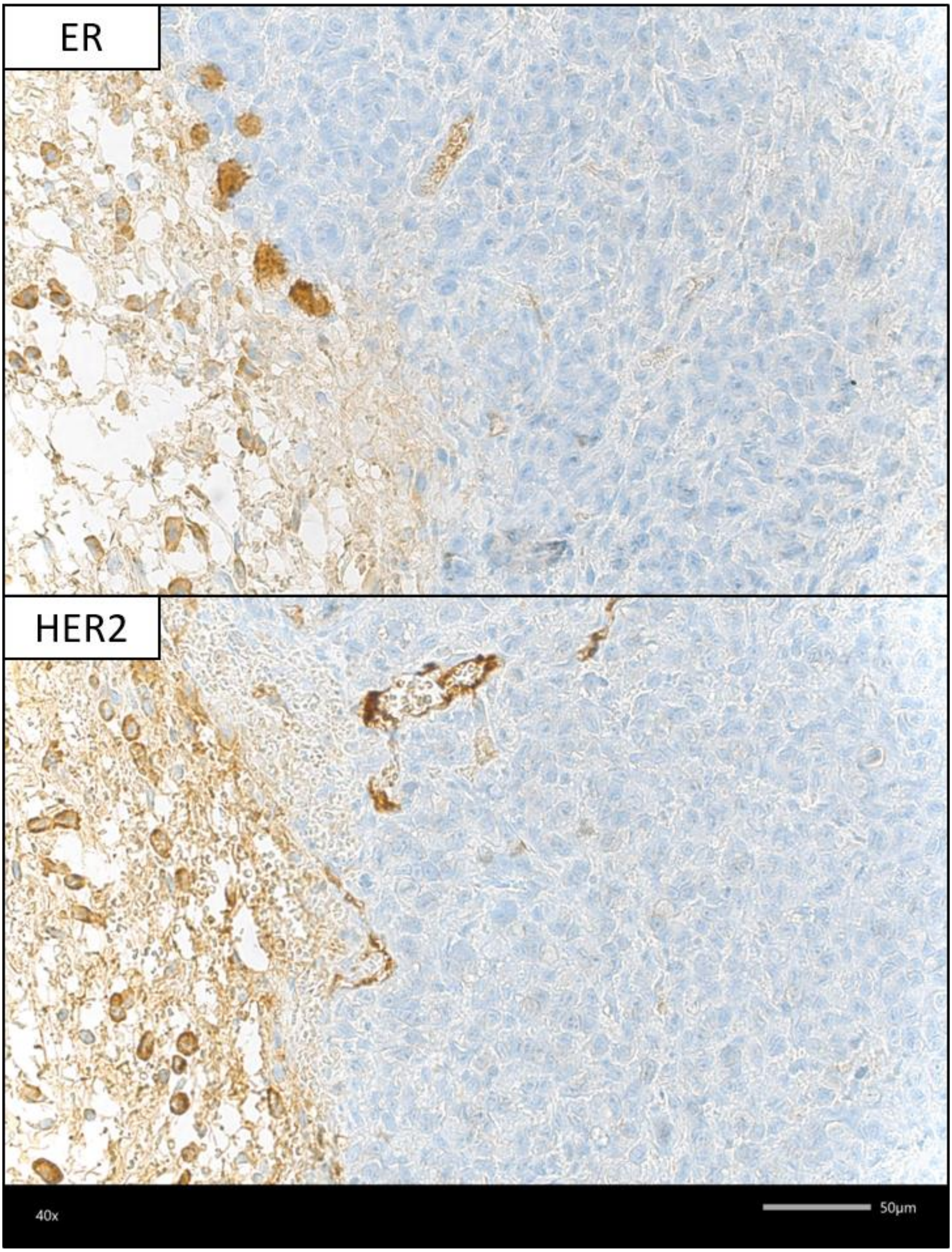

Figure S4: Higher magnification of nonspecific background staining of estrogen receptor (ER) and HER2 IHC in MDA-MB-231 mouse xenografts. Whereas cell debris, macrophages, and some soft tissue structures show unspecific staining, tumor cells do not show any specific nuclear ER staining nor specific membraneous HER2 staining, i.e., ER and HER2 status were assessed as both negative (each, x400 magnification). Scale bar: 50 µm.

Supplemental Figure S5

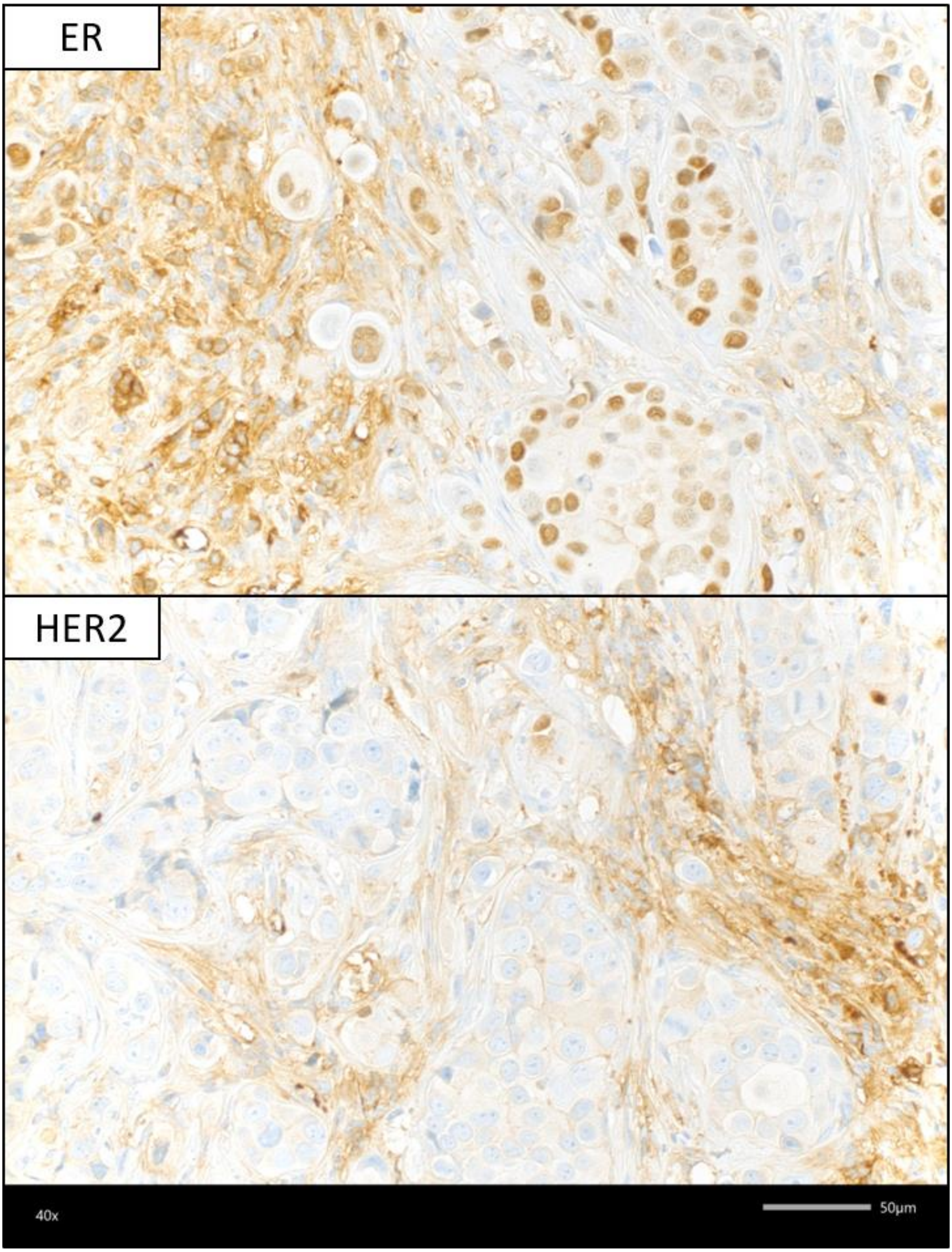

Figure S5: Higher magnification of nonspecific background immunohistochemical staining of estrogen receptor (ER) and HER2 in MCF-7 mouse xenografts. Whereas immune cells, cell debris, and soft tissue structures show unspecific staining, tumor cells show any a) specific nuclear ER staining in most of the tumor cells and b) a weak but not circumferential membranous staining (IHC score 1+, i.e., negative HER2 status) (each, x400 magnification). Scale bar: 50 µm.
